# Genu Valgum.—I

**Published:** 1891-09-26

**Authors:** 


### GENU VALGUM.—I.

Cases of knock knee may be conveniently divided into two main classes, those which arise during early childhood and those which develop during the rapidly growing period between the ages of ten and twenty.

It is well that this division should be recognised, because the treatment to be adopted is approached from a different standpoint in each case. In the first series the deformity exists in a child with bones that are soft and pliable in their entirety, whilst in the others the bones have to a greater or less extent become consolidated.

The mechanical and other causes at work in the production of knock knee have been most carefully investigated by many observers during recent years, and each and every explanation has been published with the support of sound evidence and undoubted clinical facts, but it seems to us, after a careful perusal of the subject, that each individual case can have its own explanation. If bones, ligaments, and muscles be perfectly sound and healthy there cannot well be any deformity produced; but given bones that are easily pliable, ligaments that are weak and easily relaxed, and muscles feeble in their power, either collectively or singly, then we have a condition of things when the mechanical forces brought to bear upon those structures may lead to perverted direction of pressure and consequent deformities.

The excessively rickety infant held firmly to the side of its mother in nursing may have knock knee produced in one limb and bow leg in the other before ever the child walks; and when the superincumbent weight of the child's body is brought to bear on its already distorted limbs it is easily to be understood how the deformity may increase.

If the rickety outward curve of the femur happens to be a sharp one in the lower half of that bone, the articular face of the condyles looks slightly inwards towards the middle line, and in order for the foot to be level on the ground, there must of necessity be an obtuse angle on the inner side of the knee. Again, a child with weak relaxed ligaments of the foot has no proper plantar arch, the well-known position of spurious *talipes valgus* is assumed as a matter of course, the inner malleolus overhangs, and the greater portion of the counter-vertical pressure is brought to bear upon the external condyle through the outer facet of the tibia—its growth is relatively retarded—the inner condyle relatively descends and the result is obvious. A well marked outward bend of the tibia starting at the junction of the upper epiphysis with the shaft, leads naturally to the same deformity; in fact to this, coupled with an excessive development of the inner head of the tibia, Noble Smith considers that most cases of knock knee are due.

In the second class of cases, viz., those occurring after early childhood, we must search for causes which have been at work, which were not present in earlier life, such as carrying heavy weights, much and prolonged standing, illness, or anything leading to the production of flat-foot or relaxation of the knee ligaments or the irregular relative development of the two condyles of the femur. After all, it may be said, the parents do not bring a child to us to be told how the deformity was produced, but they bring it for the deformity to be rectified. True, but it is most essential to be fully cognisant of these various mechanics of production in order to give the soundest advice as to the most appropriate treatment, and it is none the less true in this complaint than in any other that a proper understanding of its pathology is half the battle of getting it well.

Wright, of Manchester, in his book on "Children's Diseases," very properly says that "the degree of deformity, the age of the patient, and the state of the disease, whether stationary or getting worse, and the amount of care that can be bestowed upon the child, are the points to be considered in the treatment of these cases." To put this into other words we should ask ourselves the question in each individual case, "Can this deformity be corrected without operation?"

